# Nutrient intakes from complementary foods are associated with cardiometabolic biomarkers among undernourished Peruvian children

**DOI:** 10.1017/jns.2023.66

**Published:** 2023-07-19

**Authors:** Gwenyth O. Lee, Laura E. Caulfield, Maribel Paredes-Olortegui, Pablo Penataro-Yori, Mery Sigas Salas, Margaret N. Kosek

**Affiliations:** 1 Rutgers Global Health Institute and Department of Biostatistics and Epidemiology, School of Public Health, Rutgers University, New Brunswick, NJ, USA; 2 Department of International Health, Johns Hopkins Bloomberg School of Public Health, Baltimore, MD, USA; 3 Asociación Benéfica PRISMA, Iquitos, Peru; 4 University of Virginia Division of Infectious Diseases and International Health, Charlottesville, VA, USA

**Keywords:** Cardiometabolic biomarkers, Complementary foods, Nutrient intake, Peru, Stunting, HAZ, height-for-age *Z*-score, based on the WHO reference standard, HDL-c, high-density lipoprotein cholesterol, HDLZ, sd of mean HDL-c, based on the distribution of the sample, HOMA-IR, homeostatic model assessment-insulin resistance, INZ, sd of insulin, based on the distribution of the sample, LDC-c, low-density lipoprotein cholesterol, MAPZ, sd of mean arterial blood pressure, based on the distribution of the sample, MFP, meat, fish or poultry, PCA, principal components analysis, RRR, reduced rank regression, TC, total cholesterol, TG, triglycerides, TGZ, sd of triglycerides, based on the distribution of the sample, WAZ, weight-for-age *Z*-score, based on the WHO reference standard, WHZ, weight-for-height *Z*-score, based on the WHO reference standard, vLDL-c, very low-density lipoprotein cholesterol

## Abstract

Relatively little is known about how the diet of chronically undernourished children may impact cardiometabolic biomarkers. The objective of this exploratory study was to characterise relationships between dietary patterns and the cardiometabolic profile of 153 3–5-year-old Peruvian children with a high prevalence of chronic undernutrition. We collected monthly dietary recalls from children when they were 9–24 months old. At 3–5 years, additional dietary recalls were collected, and blood pressure, height, weight, subscapular skinfolds and fasting plasma glucose, insulin and lipid profiles were assessed. Nutrient intakes were expressed as average density per 100 kcals (i) from 9 to 24 months and (ii) at follow-up. The treelet transform and sparse reduced rank regress'ion (RRR) were used to summarize nutrient intake data. Linear regression models were then used to compare these factors to cardiometabolic outcomes and anthropometry. Linear regression models adjusting for subscapular skinfold-for-age *Z*-scores (SSFZ) were then used to test whether observed relationships were mediated by body composition. 26 % of children were stunted at 3–5 years old. Both treelet transform and sparse RRR-derived child dietary factors are related to protein intake and associated with total cholesterol and SSFZ. Associations between dietary factors and insulin were attenuated after adjusting for SSFZ, suggesting that body composition mediated these relationships. Dietary factors in early childhood, influenced by protein intake, are associated with cholesterol profiles, fasting glucose and body fat in a chronically undernourished population.

A nutritious childhood diet is necessary for appropriate growth and development. Dietary patterns established early in childhood set the stage for diets in later older childhood^(1)^. They may also impact cardiometabolic risk through excess weight gain, or through alterations in metabolic pathways established during key periods of developmental plasticity^(2)^.

Most reports on the relationship between diet and cardiometabolic risk factors in young children come from high-income country (HIC) populations, where the risk of child overweight and obesity outweighs the risk of chronic undernutrition. Relatively less is known about how the diet of chronically undernourished children may impact their long-term cardiometabolic health. Severe deprivation and acute undernutrition are consistently associated with cardiovascular disease, impaired glucose metabolism and the metabolic syndrome later in life^(3)^. Stunting, resulting from poorer growth in infancy and early childhood, has also been associated with an increased risk of adult diabetes or cardiovascular disease^(4–7)^, possibly through impairments to biological pathways involving metabolic regulation and insulin signalling^(8)^. Thus, it is important to understand the role of early dietary patterns on cardiometabolic risk factors in settings where the early diet increases risk or protects children from chronic undernutrition.

Among children at risk of overweight and obesity, the consumption of specific foods and nutrients, such as greater consumption of vegetables and a lower intake of sugars, have been associated with lower body weight^(9–11)^, as have adherence to Mediterranean and what are considered anti-inflammatory dietary patterns^(12,13)^. Sugar consumption has been consistently associated with adverse cardiometabolic profiles^(14–16)^, whereas adherence to ‘healthy’ dietary patterns^(17,18)^ and high-quality, high-fibre carbohydrate consumption^(19)^ are typically associated with better cardiometabolic indicators. However, the body of literature emerging from chronically undernourished populations is much more limited. In this context, there is some evidence linking protein consumption in infancy and childhood with higher body fat^(20)^ and increased adult adiposity^(21)^, although this translated into protection from diabetes, rather than an increased risk^(21)^. These findings are supported by studies from HICs which have found that protein intake, and the ratio of animal to plant protein, are negatively associated with diverse components of cardiometabolic health^(22,23)^. In HICs, positive, negative and null associations have been reported between protein intake in infancy and cardiometabolic risk factors such as blood pressure, triglycerides and serum insulin^(1,24,25)^.

This lack of consistency may derive from the fact that the relationship between specific nutrients and cardiometabolic risk is complex, and nutrients are often consumed in relation to other aspects of diet, which may be risk-promoting or protective. This limitation, which is common to dietary data analysis, has led an increasing preference on the part of researchers towards analysing diets holistically. Dietary pattern analyses, both *a priori* (hypothesis-driven) and *a posteriori* (data-driven), accomplish this aim. Although well-established methods for dietary pattern analysis such as principal components analysis remain popular, several newer methods, including the treelet transform and reduced rank regression (RRR)^(26)^, are also gaining ground to inform understandings of the complex relationship between diet and health.

We previously reported associations between biomarkers of gut function related to chronic enteropathogen exposure and the cardiometabolic profile of 36–60-month-old Peruvian children^(27)^. In the current exploratory study, we characterise the relationship between dietary patterns in infancy (9–24 months) and childhood (36–60 months), and the childhood cardiometabolic profile.

## Methods

### Study design and study population

The study was nested within the Peru cohort of the ‘Etiology, Risk Factors and Interactions of Enteric Infections and Malnutrition and the Consequences for Child Health and Development (MAL-ED)’ study. MAL-ED was a prospective, longitudinal birth cohort study to evaluate the relationship between enteric and nutritional exposures and child physical growth, cognitive development and immune response^(28,29)^. The characteristics of the population^(29)^ and the overall design of the MAL-ED cohort study^(28)^ have previously been reported. Briefly, 303 neonates were enrolled with the goal of following at least 200 infants through 24 months of age, and then subsequently to 60 months^(28,29)^. Healthy singleton newborns weighing at least 1500 g, aged <17 d at recruitment, generally healthy, with no siblings already participating in the study, and born to a mother at least 16 years of age able to give informed consent and who planned to remain in the community for the next 6 months, were eligible to enrol. Enrolment was completed between December 2009 and February 2012. Between February and April 2015, when the children were 36–60 months of age, we conducted a study to examine cardiometabolic profiles. Only children 36–60 months of age who participated in the original MAL-ED cohort study through at least 18 months of age and were still living in the community or in Iquitos city were eligible to participate. The protocol for this nested study was explained to parents, who provided written informed consent if they decided to participate.

The characteristics of this cohort have previously been described. The mean length-for-age *Z*-score was −1⋅87 at 24 months of age and −1⋅44 at the time of the follow-up study^(27)^. Descriptive information about the diet of participating children has also been comprehensively documented^(30–32)^. Briefly, macronutrient intakes, including protein intake, were generally sufficient. The probability of dietary adequacy for specific micronutrients and minerals varied by nutrient. For instance, the probability of dietary adequacy for vitamin D was very low, while the probability of adequacy for niacin and vitamin B6 varied according to age-specific cut-offs^(32)^.

#### Dietary data collection

Beginning at enrolment and continuing through 24 months, information on breast-feeding practices were obtained through twice weekly house visits. Monthly from 1 to 8 months, a questionnaire was also administered to collect additional information on feeding practices. Beginning at 9 months and monthly through 24 months, a 24-h dietary recall was administered to quantify energy, macro- and micronutrient intakes (‘Infant’ dietary data)^(33)^. During the MAL-ED follow-up of children to 60 months, dietary recalls were also collected monthly at the Peru site. Here, we use three dietary recalls collected over a 1-month period to characterise diet at the age at which each child's cardiometabolic profile was determined (‘child’ dietary data).

#### Cardiometabolic profile

Parents were asked to fast their children overnight before the visit, which was conducted in early morning (between 5 am and 9 am). At the study visit, a fasting blood sample was taken, and blood pressure was measured three times for each participant, using a manual sphygmomanometer with an appropriately sized child cuff and waiting at least 1 min between each measurement^(34)^. Anthropometric measurements were taken by trained personnel, including height, weight, waist circumference and subscapular skinfold thickness^(35)^.

Fasting plasma glucose and insulin concentrations were determined as well as a lipid profile. Glucose, triglycerides (TG), total cholesterol (TC), high-density lipoprotein cholesterol (HDL-c), low-density lipoprotein cholesterol (LDL-c) and very low-density lipoprotein cholesterol (vLDL-c) concentrations were determined using an enzymatic colorimetric method on an automated Cobas c 311 clinical chemistry analyzer. Insulin was determined using an electrochemiluminescence immunoassay on a Cobas E411 immunoassay analyzer.

Child anthropometry (subscapular skinfold-for-age *Z*-score, height-for-age *Z*-score, weight-for-age *Z*-score and weight-for-height *Z*-score (WHZ)) were calculated using the WHO child growth standards^(36,37)^. Each child's breast-feeding history was summarised as the total prevalence of any breast-feeding from 0 to 6 months of age and from 9 to 24 months of age. Socioeconomic status (SES) was summarised by the Water/sanitation, Assets, Maternal education and Income (WAMI) index, a socioeconomic score that was developed and evaluated for validity by Psaki *et al.*^(38)^ for the MAL-ED study and includes indicators of water sanitation and hygiene, assets, household size, dwelling size, maternal education and income. The score ranges from 0 to 1 and, therefore, its coefficient in each model can be interpreted as the association of SES on the outcome of interest comparing the poorest possible and the greatest possible SES score.

#### Statistical analysis

For analyses, macro- and micronutrient intakes were expressed as nutrient densities, then averaged across all recalls. Results are therefore expressed as average nutrient density per 100 kcal between (i) 9–24 months of age and (ii) at the time of the cardiometabolic profiling when children were between 36 and 60 months of age. For ease of interpretation and in line in other reports^(14)^, we considered protein and iron intakes as protein from meat, fish or poultry (MFP) or non-MFP source protein. We also considered intakes by type of fat: saturated fat, monounsaturated fat and polyunsaturated fats. Carbohydrates were further identified as ‘sugar’ or ‘non-sugar’ carbohydrates.

To understand the dietary sources of each nutrient, we assigned each food consumed to one of thirteen categories: coffee or tea, grains, roots, legumes, yellow fruits, other fruits and vegetables, organ meats, eggs, fish, dairy, poultry, other meats (e.g. beef and pork) or sweets. We used bar graphs to visualise the relative proportion of each nutrient intake that derived from each of these groups. For example, what percentage of protein intake came from organ meats, eggs, fish, poultry or other sources.

To identify dietary patterns associated with cardiometabolic risk factors among young Peruvian children, we used two methods. Given that relatively little is known about how dietary patterns in infancy may relate to cardiometabolic biomarkers, and because of the large number of cardiometabolic biomarker outcome variables available to us, we conducted an exploratory analysis of potential associations that is intended to be hypothesis-generating, rather than confirmatory. Our primary method of analysis was the treelet transform, an *a posteriori* approach that develops dietary factors based on hierarchical clustering of naturally grouped subsets of the original data^(39,40)^. Like principal component analysis (PCA), the treelet transform derives factors based on relationships among dietary variables and does not integrate information about the relationship between these factors and the outcome of interest. However, unlike PCA, not all dietary components are included in the final factor or factors^(39,40)^. To implement the treelet transform, we identified the cut-level for the associated cluster tree by cross-validation and used scree plots to identify the appropriate number of factors. The treelet transform is closely related to PCA, as it uses a correlation matrix to produce factors in the same manner as PCA. Therefore, for comparison, parallel PCA analyses were also performed.

To complement this primary approach, our second method used RRR to identify the combinations of nutrient intakes that explain the most variation in the cardiometabolic outcomes^(17,40,41)^. RRR is also *a posteriori* approach intended to uncover relationships between complex dietary patterns and disease outcomes^(17,24,26,42)^. RRR is often presented as a complement to the treelet transform and PCA, because these two methods aim to maximise variation in the dietary intake of the study population, whereas RRR maximises variations in the diet associated with disease outcomes, even if these aspects are evenly distributed across the population. However, RRR is vulnerable to collinearity among the predictor variables^(43)^, a typical feature of dietary data, and RRR may be prone to influence by chance findings^(44)^, making it is important to assess the stability of RRR-derived patterns. Therefore, we used sparse RRR, an extension to RRR that reducing the number of dietary variables contributing to the RRR model, to increase stability^(44)^.

RRR is hypothesis-driven, because response variables are selected *a priori*, and dietary patterns are constructed to maximise the associations with these variables. Therefore, some cardiometabolic biomarkers were used within the RRR, and the derived dietary pattern was then tested for associations with other biomarkers, using bivariate regression models. The outcomes we selected for the RRR were: subscapular skinfold-for-age *Z*-score based on the WHO child growth standards (SSFZ)^(37)^, as well as the sd of mean arterial blood pressure (MAPZ), HDL-c (HDLZ), triglycerides (TGZ) and insulin (INZ) based on the distributions of each outcome in the sample. The derived dietary pattern was then related to systolic blood pressure, diastolic blood pressure, TC, low-density lipoprotein cholesterol (LDL-c), very low-density lipoprotein (vLDL-c), glucose, HOMA-R, HAZ, WAZ and WHZ) were considered.

Because infant dietary factors may influence the cardiometabolic profile in a distinct manner from the child diet, infant and child dietary data were analysed separately. After developing bivariate models to test the association between each dietary factor and cardiometabolic outcome, we then developed multivariate models that adjusted for SSFZ. The purpose of this adjustment was to determine whether observed associations were mediated by body composition. We also considered an adjustment for age, sex, breast-feeding from 9–24 months of age, defined as the percent of days with any breastmilk intake during that period. *P*-values of less than or equal to 0⋅05 were taken to represent statistical significance.

## Results

The characteristics of the study children are reported in Table 1. Of 303 children initially enrolled in the MAL-ED cohort, at the time of cardiometabolic profiling 3 had withdrawn from the study; 15 were ineligible because they were >5 years old and 3 had passed away. Among the remainder, seventy-six were ineligible for cardiometabolic profiling because family mobility had resulted in significant gaps in data collected while they were between 0 and 18 months old; and forty-two were eligible but were no longer living in the study catchment area. A total of 164 children were invited to participate in the sub-study and 156 (95 %) accepted.

**Table 1.** Characteristics of the study population

	*N*; Mean (sd)
*N*	153
Age (months)	48⋅4 (7⋅5)
Systolic blood pressure (mmHg)	81⋅6 (11⋅2)
Diastolic blood pressure (mmHg)	55⋅2 (8⋅7)
Mean arterial pressure	64⋅0 (8⋅5)
Triglycerides (mmol/l)	0⋅98 (0⋅38)
Total cholesterol (mmol/l)	3⋅66 (0⋅52)
High-density lipoprotein (mmol/l)	1⋅15 (0⋅23)
Low-density lipoprotein (mmol/l)	2⋅17 (0⋅45)
Very low-density lipoprotein (mmol/l)	
Glucose (mmol/l)	4⋅58 (0⋅43)
Insulin (μIU/ml)	4⋅3 (2⋅6)
Homeostatic model assessment-insulin resistance	0⋅9 (0⋅6)
Subscapular skinfold-for-age *Z*	−0⋅09 (1⋅05)
Height-for-age *Z*	−1⋅45 (1⋅05)
Weight-for-age *Z*	−0⋅54 (0⋅97)
Weight-for-height *Z*	0⋅48 (1⋅17)

**Fig. 1.**
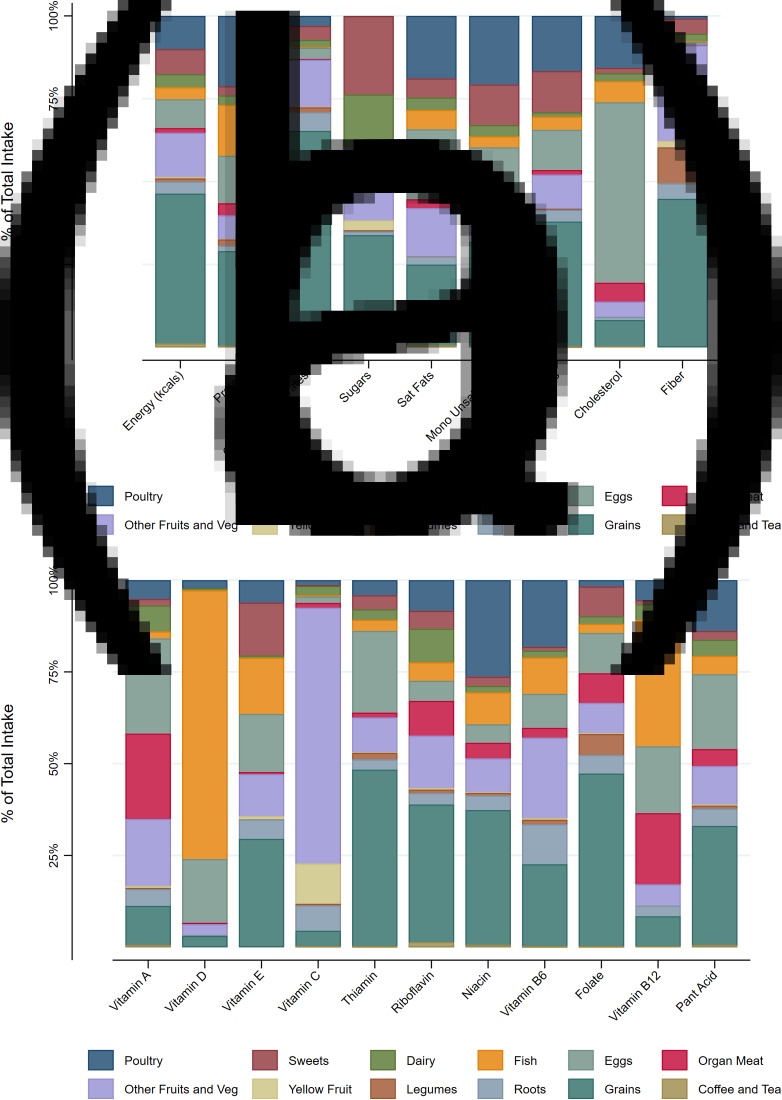
Relative food group contributions to total nutrient intake.

Of the 156 children who participated in the sub-study, 3 were excluded for having 5 or fewer recalls from 9 to 24 months. The children included in the analysis had 11–17 recalls available from 9 to 24 months and between 1 and 3 recalls available at follow-up (7 children had only 1 recall, 11 children had 2 recalls and 135 had 3 recalls). The food categories contributing to intake of each nutrient are shown in Fig. 1. Nutrient intake estimates are shown in Table 2. There were no associations between any outcome variable and breast-feeding, where breast-feeding was defined as the percent of days with any breastmilk from 0 to 9 and 9 to 24 months of age.

**Table 2.** Estimated daily dietary intake at 9–24 months (infant) and 3–5 years old (child)

	Infant	Child	
% of days with any breastmilk	97⋅8 %	n/a	
# Recalls per child	11–17	1–3	
	**Mean (sd)**	**Mean (sd)**	**As a percentage of total energy intake**
Energy (kcal)	725 (183)	1158 (248)	
MFP Protein (g day per 100 kcal)	0⋅94 (0⋅41)	1⋅05 (0⋅60)	10⋅4 % (2⋅0 %)
Non-MFP Protein (g per 100 kcal)	1⋅73 (0⋅31)	1⋅54 (0⋅30)	
Saturated fat (g per 100 kcal)	1⋅05 (0⋅26)	1⋅11 (0⋅32)	20⋅4 % (5⋅1 %)
Monounsaturated fat (g per 100 kcal)	0⋅63 (0⋅15)	0⋅65 (0⋅19)	
Polyunsaturated fat (g per 100 kcal)	0⋅32 (0⋅08)	0⋅35 (0⋅13)	
Non-sugar carbohydrates (g per 100 kcal)	9⋅58 (1⋅25)	9⋅73 (1⋅52)	68⋅7 % (6⋅2 %)
Sugar (g per 100 kcal)	8⋅09 (1⋅54)	7⋅74 (1⋅80)	
Cholesterol (mg per 100 kcal)	16⋅90 (6⋅27)	16⋅17 (7⋅33)	-
			**% above Estimated Average Requirement (EAR)**
Fibre (g per 100 kcal)	0⋅28 (0⋅09)	0⋅30 (0⋅18)	0⋅6 %
Vitamin A (μg per 100 kcal)^a^	38⋅6 (25⋅6)	19⋅5 (10⋅9)	45⋅1 %
Vitamin D (μg per 100 kcal)^a^	0⋅28 (0⋅18)	0⋅31 (0⋅26)	22⋅9 %
Vitamin E (mg per 100 kcal)^a^	0⋅13 (0⋅04)	0⋅14 (0⋅05)	0⋅0 %
Vitamin C (mg per 100 kcal)^a^	11⋅06 (9⋅73)	11⋅87 (20⋅30)	64⋅1 %
Thiamine (mg per 100 kcal)^a^	0⋅04 (0⋅02)	0⋅04 (0⋅01)	25⋅5 %
Riboflavine (mg per 100 kcal)^a^	0⋅07 (0⋅03)	0⋅05 (0⋅02)	52⋅3 %
Niacin (mg per 100 kcal)^a^	0⋅56 (0⋅10)	0⋅58 (0⋅14)	39⋅2 %
Vitamin B6 (mg per 100 kcal)^a^	0⋅05 (0⋅01)	0⋅05 (0⋅02)	56⋅9 %
Folate (mg per 100 kcal)^a^	8⋅73 (3⋅01)	6⋅94 (1⋅95)	1⋅3 %
Vitamin B12 (mg per 100 kcal)^a^	0⋅16 (0⋅10)	0⋅11 (0⋅06)	65⋅4 %
Pantothenic acid (mg per 100 kcal)^a^	0⋅25 (0⋅05)	0⋅22 (0⋅04)	49⋅0 %
Calcium (mg per 100 kcal)^a^	32⋅68 (17⋅93)	22⋅64 (10⋅04)	3⋅9 %
Phosphorus (mg per 100 kcal)^d^	46⋅82 (10⋅26)	43⋅84 (8⋅43)	75⋅2 %
Magnesium^d^	10⋅80 (1⋅79)	10⋅23 (2⋅22)	89⋅5 %
MFP Iron (mg per 100 kcal)^a^^,^^b^	0⋅10 (0⋅06)	0⋅09 (0⋅07)	26⋅1 %
Non-MFP Iron (mg per 100 kcal)^a^^,^^b^	0⋅52 (0⋅32)	0⋅37 (0⋅14)	
Zinc (mg per 100 kcal)^a^^,^^c^	0⋅31 (0⋅06)	0⋅31 (0⋅06)	19⋅0 %
Copper (mg per 100 kcal)^e^	0⋅04 (0⋅01)	0⋅04 (0⋅01)	0⋅0 %
Manganese (mg per 100 kcal)^e^	0⋅19 (0⋅16)	0⋅15 (0⋅06)	55⋅6 %

a Dietary reference intakes (DRIs) for vitamin A, vitamin D, vitamin E, vitamin D, thiamine, riboflavine, niacin, vitamin B6, folate, vitamin B12, pantothenic acid and calcium from WHO/FAO (2004)^(50)^.

b Assuming 10 % bioavailability^(50)^.

c Assuming moderate bioavailability^(50)^.

d DRIs for phosphorus and magnesium are from dietary reference intakes^(51)^.

e DRIs for copper and manganese are from dietary reference intakes^(52)^.

(*N* 153).

**Fig. 2.**
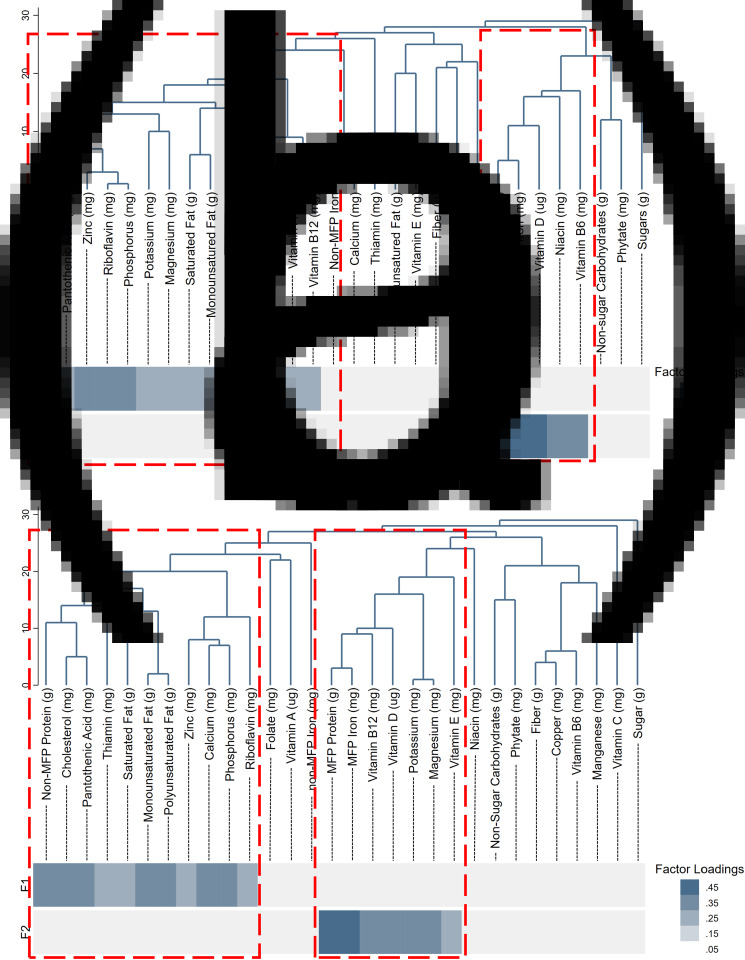
Treelet transform-derived infant and child dietary factors.

Treelet transform-derived infant and child nutrient intake patterns are illustrated in Figs. 2(a) and 2(b). As others have noted^(26)^, treelet transform results were intuitive, as nutrients with similar dietary sources were grouped together. Dendrograms were also useful for visualising the clustering process. Dendrograms for infant and child nutrient intake data were produced separately but resulted in similar patterns. In both cases, the treelet transform each extracted two factors with similar interpretation. The first factor, ‘Non-MFP Protein’, was associated with a higher consumption of non-MFP protein as well as both saturated and monounsaturated fats, and cholesterol. The second factor, ‘MFP Protein’, was associated with a higher consumption of MFP Protein, MFP Iron, vitamin D, niacin and vitamin B6. In both infant and child data, the first factor was positively associated with SES as represented by the WAMI score, whereas the second factor was not associated with SES. The first infant dietary factor was 1⋅65 standard deviations higher among children with the highest *v*. the lowest possible WAMI score (95 % CI 0⋅41, 2⋅90); and the first child dietary factor was 1⋅38 standard deviation higher factor for children with the highest *v*. the lowest possible WAMI score (95 % CI 0⋅15, 2⋅62).

**Fig. 3.**
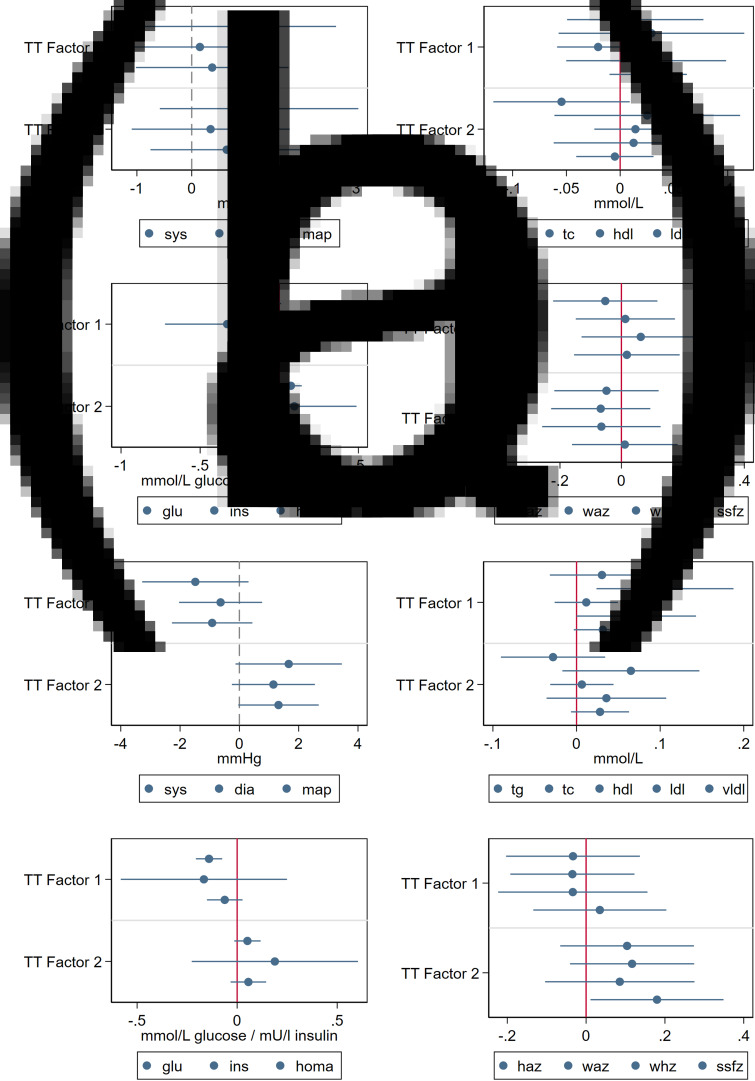
Associations between treelet transform-derived dietary factors and cardiometabolic biomarkers.

No associations were found between the infant dietary patterns and cardiometabolic biomarkers, except that the second infant dietary factor, which was composed of MFP protein and iron and was negatively associated with fasting plasma glucose (Fig. 3(a)). The first child dietary factor was associated with higher fasting TC and HDL-c and lower fasting plasma glucose, whereas the second child dietary factor was associated with higher SSFZ and higher mean arterial pressure (Fig. 3(b)). The treelet transform-derived factors were highly correlated with PCA-derived factors and had similar associations with cardiometabolic outcomes (presented in Supplementary Figs. S1 and S2).

**Fig. 4.**
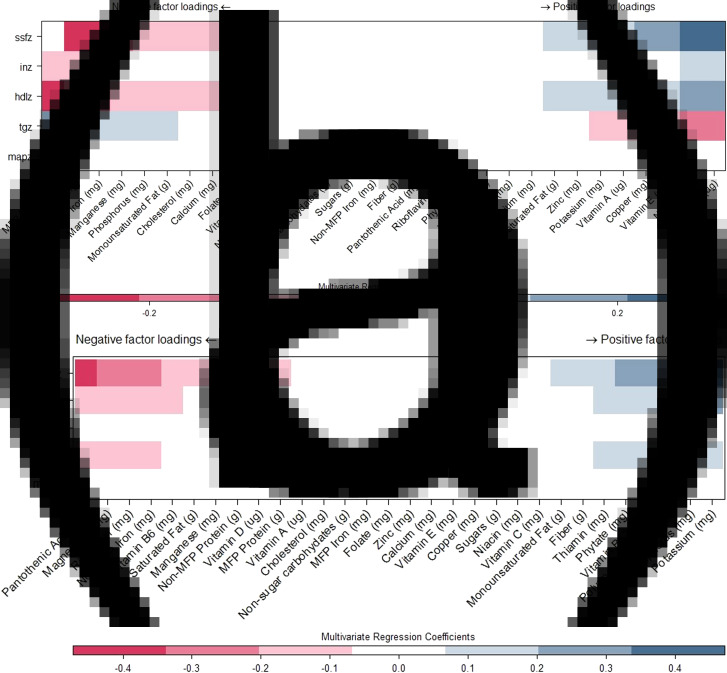
Sparse RRR-derived infant and child dietary factors.

Sparse RRR extracted one factor for both infant and child dietary patterns. The sparse RRR-derived factor explained 6⋅2 % of the variation in the response variables for infant dietary data, and 6⋅8 % of the variation in the response variables for child dietary data. The infant dietary factor had negative factor loadings for MFP protein and MFP iron, monounsaturated fat, cholesterol and calcium and positive factor loadings for vitamin D, niacin and polyunsaturated fat (Fig. 4(a)). For the child dietary patterns, pantothenic acid (correlated with cholesterol intake) and non-MFP protein had negative factor loadings, whereas potassium, phosphorus and polyunsaturated fat had the highest positive loadings (Fig. 4(b)). There was no association between the infant dietary pattern and household SES (0⋅25 standard deviation higher factor for children with the highest *v*. the lowest possible WAMI score (95 % CI −0⋅36, 0⋅87), while the child factor was positively associated with household SES (1⋅08 standard deviation higher factor for children with the highest *v*. the lowest possible WAMI score (95 % CI 0⋅48, 1⋅68). Figs. 4(a) and 4(b) also demonstrate the nutrients contributing to each of the two final factors according to their association with each cardiometabolic biomarker included in the RRR. For example, in the infant data, MFP protein intake was negatively associated with fasting insulin and fasting HDL-c, and positively associated with TGs; as a result, the final infant RRR-derived factor had a negative loading for MFP protein.

**Fig. 5.**
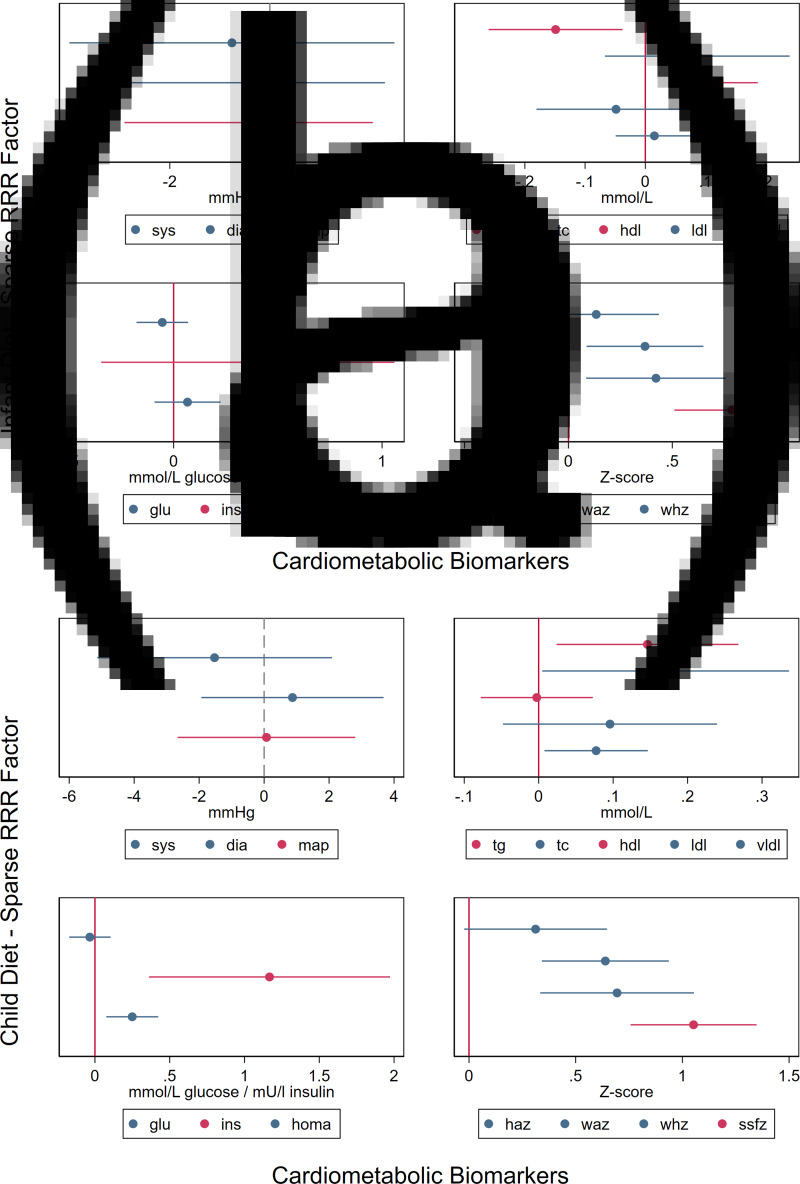
Associations between sparse RRR-derived infant and child dietary factors and cardiometabolic biomarkers. Red bars indicate cardiometabolic biomarkers utilised to generate RRR-derived factors; blue bars indicate biomarkers that were not used in the RRR.

RRR is hypothesis-driven, because response variables are selected *a priori*, and dietary patterns are constructed to maximise the associations with these variables. Therefore, some cardiometabolic biomarkers (SSFZ, MAPZ, HDLZ, TGZ and INZ) were used in the RRR, and the derived dietary pattern was then tested for associations with other biomarkers. The RRR-derived infant dietary factor was associated with WAZ and WHZ, while the RRR-derived child factor was also associated with TC, vLDL-c, HOMA-IR, WAZ and WHZ. Regression coefficients to test associations between each dietary pattern and cardiometabolic outcome are displayed in Figs. 5(a) and 5(b). Finally, for child RRR dietary patterns, we note that associations with insulin and HOMA-IR were attenuated after adjusting for SSFZ, suggesting that body composition mediated these relationships. In contrast, associations between dietary patterns, TG, TC and vLDL were not attenuated by SSFZ (Table 3).

**Table 3.** Attenuation of diet–cardiometabolic outcomes on adjustment for subscapular skinfold-for-age *Z*-score

	TG	TC	HDL	vLDL	Insulin	HOMA-IR	WAZ	WHZ
Infant RRR Factor 1								
Unadjusted	−0⋅15 (−0⋅6, −0⋅04)		0⋅12 (0⋅06, 0⋅19)				0⋅37 (0⋅09, 0⋅65)	0⋅42 (0⋅08, 0⋅76)
Adjusted	−0⋅20 (−0⋅32, −0⋅08)		0⋅15 (0⋅08, 0⋅22)				−0⋅13 (−0⋅38, 0⋅11)	−0⋅10 (−0⋅42, 0⋅21)
Percent attenuation	+33 %	−	+45 %	−	−	−	−65 %	−123 %
Child RRR Factor 1								
Unadjusted	0⋅15 (0⋅02, 0⋅27)	0⋅17 (0⋅00, 0⋅34)		0⋅08 (0⋅01, 0⋅15)	1⋅17 (0⋅36, 1⋅97)	0⋅25 (0⋅08, 0⋅42)	0⋅64 (0⋅34, 0⋅93)	0⋅69 (0⋅33, 1⋅05)
Adjusted	0⋅17 (0⋅02, 0⋅31)	0⋅18 (−0⋅01, 0⋅37)		0⋅07 (−0⋅01, 0⋅15)	0⋅52 (−0⋅39, 1⋅43)	0⋅12 (−0⋅08, 0⋅32)	0⋅02 (−0⋅26, 0⋅29)	0⋅06 (−0⋅31, 0⋅42)
Percent attenuation	+13 %	+6 %	−	+13 %	−55 %	−52 %	−97 %	−91 %

The original coefficients are also shown in Fig. 5. Here, we display the percent change in beta coefficient after adjusting for subscapular skinfold-for-age *Z*-score (SSFZ). A positive percent attenuation represents a shift away from the null and a negative attenuation value represents a shift towards the null. For example, a positive association between the RRR-derived child dietary factor and HOMA-IR was attenuated after SSFZ was included in the model.

## Discussion

Investigating dietary patterns in relation to cardiometabolic biomarkers may provide new insights into the development of later cardiometabolic diseases in a chronically undernourished population. In this exploratory study, we used treelet transform analysis and RRR, two techniques aimed at data reduction, to identify dietary factors associated with the early child cardiometabolic profile. Our results should be interpreted within the context of adequate protein intake, and cardiometabolic profiles frequently characterised by low HDL-c, but very little overweight or obesity^(27)^.

While inadequate protein growth can lead to poor growth outcomes, high protein intake, especially MFP protein, is associated with increased weight gain during infancy and correlates with later obesity^(1)^, but decreased risk of cardiometabolic disease for individuals who were stunted as children^(21)^. Protein intake is also highly related to overall dietary quality^(23)^. Our results suggest that overall protein sources in the child diet are associated with body fat, triglycerides and cholesterol profiles. These results were consistent when deriving dietary factors based on variation in the exposure (the treelet transform), and when deriving dietary factors based on variation in the outcome (RRR). An additional positive association with MFP protein and fasting plasma glucose was observed in treelet transform-derived factors. In this population, protein is sourced primarily from fresh fish, eggs and poultry, and ‘nutritional buffering’ in the form of substitution of protein sources (for example, purchasing organ meat or canned tuna instead of red meat or fresh fish) is an important food insecurity coping strategy^(45)^. As has been reported in other populations^(23)^, we observed inverse correlations between MFP and non-MFP protein, as well as associations with SES: wealthier children consumed more non-MFP protein, and similar amounts of MFP protein. Finally, adjusting for SSFZ caused associations between dietary factors, triglycerides and insulin resistance to be attenuated, whereas associations with blood pressure and cholesterol profiles were unaffected. These findings support other reports that diet in early childhood may affect cardiometabolic health through pathways that are mediated by, as well as independent of, differences in body composition^(1,17)^.

Because infant dietary factors may influence the cardiometabolic profile in a distinct manner from the child diet, infant and child dietary data were analysed separately. However, infant and child dietary patterns were correlated, which limits our ability to fully examine independent effects of diets consumed at different ages. However, the results of child diet analyses were more consistent than analyses of infant diets and later cardiometabolic outcomes. A single factor based on RRR was unassociated with cardiometabolic biomarkers (other than those used in the RRR), whereas the treelet transform-derived factor suggested associations between MFP Protein consumption and higher fasting plasma glucose. Because the results during infancy are inconsistent, we are cautious about concluding that infant diets are associated with cardiometabolic risk factors in early childhood.

We used two increasingly popular techniques for dietary pattern analysis: the treelet transform and RRR. Both methods include a variable-selection step that reduces the number of nutrients included in the final factor. We found that treelet transform produced factors that were readily interpretable compared to RRR, as nutrients were naturally grouped and could be interpreted according to their dietary source. In contrast, RRR, although appealing in its ability to derive dietary patterns based on the outcomes of interest, also presents several challenges. RRR is vulnerable to high collinearity, which can create challenges for nutrient intake data, reduce the stability of RRR-derived factors and complicate the interpretation of these factors. We addressed these limitations using sparse RRR^(46)^. The results of this RRR analysis of child dietary data were reinforced by positive associations with cardiometabolic outcomes not utilised in the initial construction of the RRR factors, as well as similar results in treelet transform-derived factors and confirmatory PCA.

Our study has several limitations, some of which we addressed through our analytic strategy. Because the population was highly mobile, only ~50 % of children initially enrolled into the study were available for recruitment at the 3–5-year follow-up visit. This creates the potential for attrition bias (e.g. if the children who left the study had associations between dietary intake and cardiometabolic outcomes that were different than those of the group observed). Although many dietary recalls contributed to the analysis, cardiometabolic outcomes were only measured once per child. Since reproducibility of some cardiometabolic measures, particularly fasting insulin^(47)^, is low, these results should be limited with greater caution. As we previously discussed, we used data reduction techniques to summarise our nutrient intake data and reduce the number of comparisons made. However, given the lack of an *a priori* hypothesis about the role of diet in the cardiometabolic development of this population, our analysis was intended to be hypothesis-generating, rather than confirmatory. Our sample size for this analysis (*N* 156) 156 provided statistical power to detect standardised effect sizes of Cohen's *d* > 0⋅5or greater: a remarkably universal cut-off for clinical significance^(48)^. As a result, the statistically significant results within this exploratory analysis are also within what is generally considered to be a plausible range for clinical significance. However, this cannot be confirmed, because, although early life cardiometabolic risk factors have been linked to adult disease risk^(49)^ relatively little is known about whether this is similar in populations such as ours. We are further limited by a lack of information about the cardiometabolic health of this population in later childhood or adolescence.

The study also has some strengths, including the nature of the study population. As previously mentioned, we benefit from very rich dietary data collected throughout infancy and early childhood^(33)^, as well as high resolution data on many other factors that impact the health of young children^(28)^. We also note that this group had a high prevalence of stunting, but not overweight, and that the most common cardiometabolic risk factor profile was distinct from the more common profile of children at risk of overnutrition in obesogenic low- or middle-income country (LMIC) and HIC environments^(27)^. Nevertheless, adult overweight and obesity is a growing concern for these children^(29)^, so the identification of early life exposures that increase adult cardiometabolic risk are highly relevant. The diet of these children is also diverse and continues to incorporate traditional Amazonian dietary staples.

In conclusion, aspects of the concurrently measured child diet related to protein intake were associated with multiple cardiometabolic biomarkers in a chronically undernourished Peruvian Amazonian population. Our results suggest multiple relationships between child dietary patterns and cardiometabolic, some mediated by, and some independent of, body fat. Further work should investigate the extent to which relationships translate into longer-term cardiometabolic risk for populations undergoing the nutritional transition.

## Supplementary material

For supplementary material accompanying this paper visit https://doi.org/10.1017/jns.2023.66. click here to view supplementary material
