# Supplementary material for: Nutrient intakes from complementary foods are associated with cardiometabolic biomarkers among undernourished Peruvian children
Source: J Nutr Sci. 2023 Jul 19;12:e80. doi: 10.1017/jns.2023.66 (PMC10388437; doi:10.1017/jns.2023.66)
Supplement: Supplementary file 1 [file S2048679023000666sup001.docx]

**Supplemental Figure 1. Participant Flow Chart**


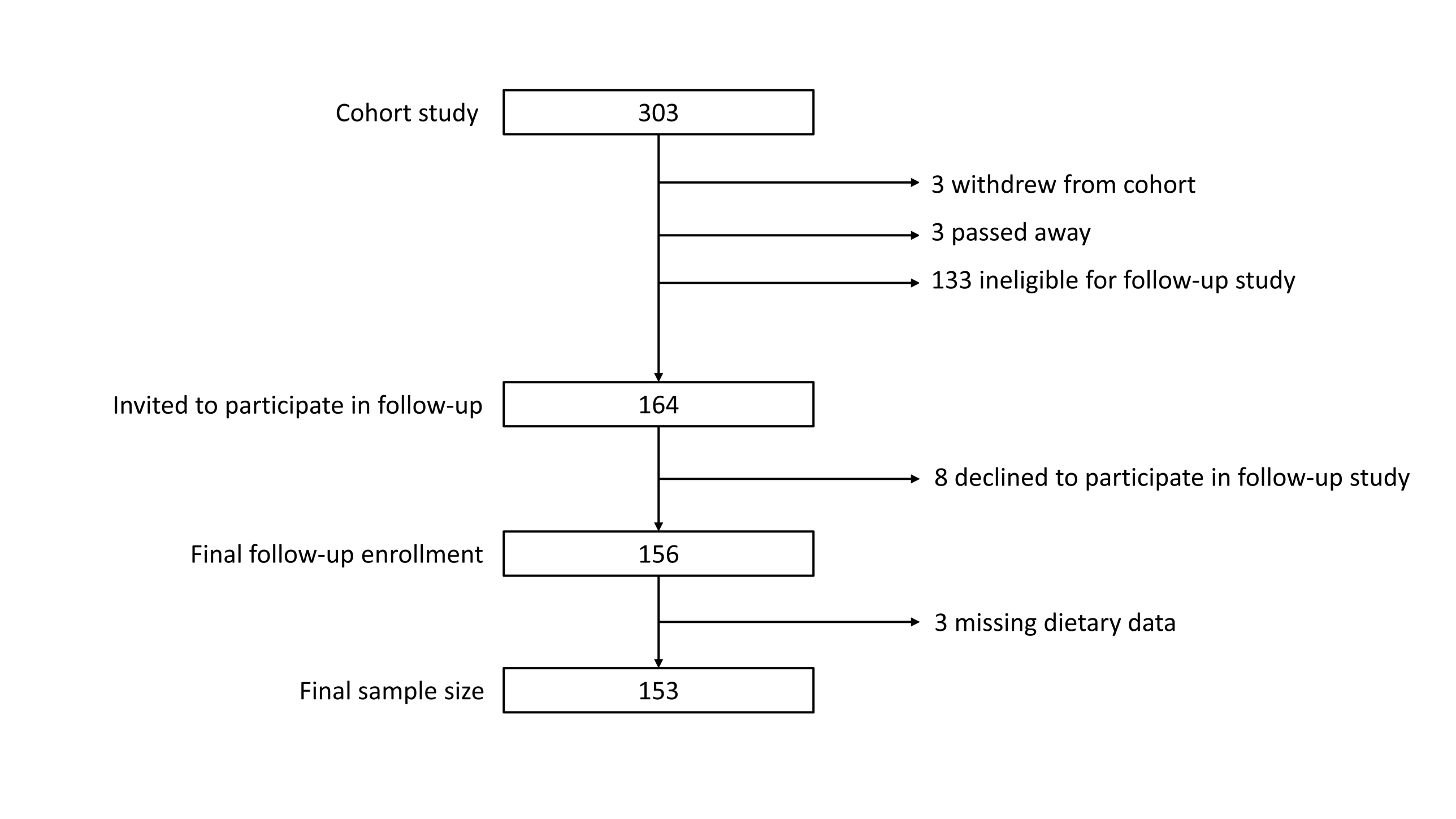


**Supplemental Figure 2.PCA-Derived Infant Dietary Factors**

| 1. **Dendrogram of PCA Dietary Factors (Infant Data)**   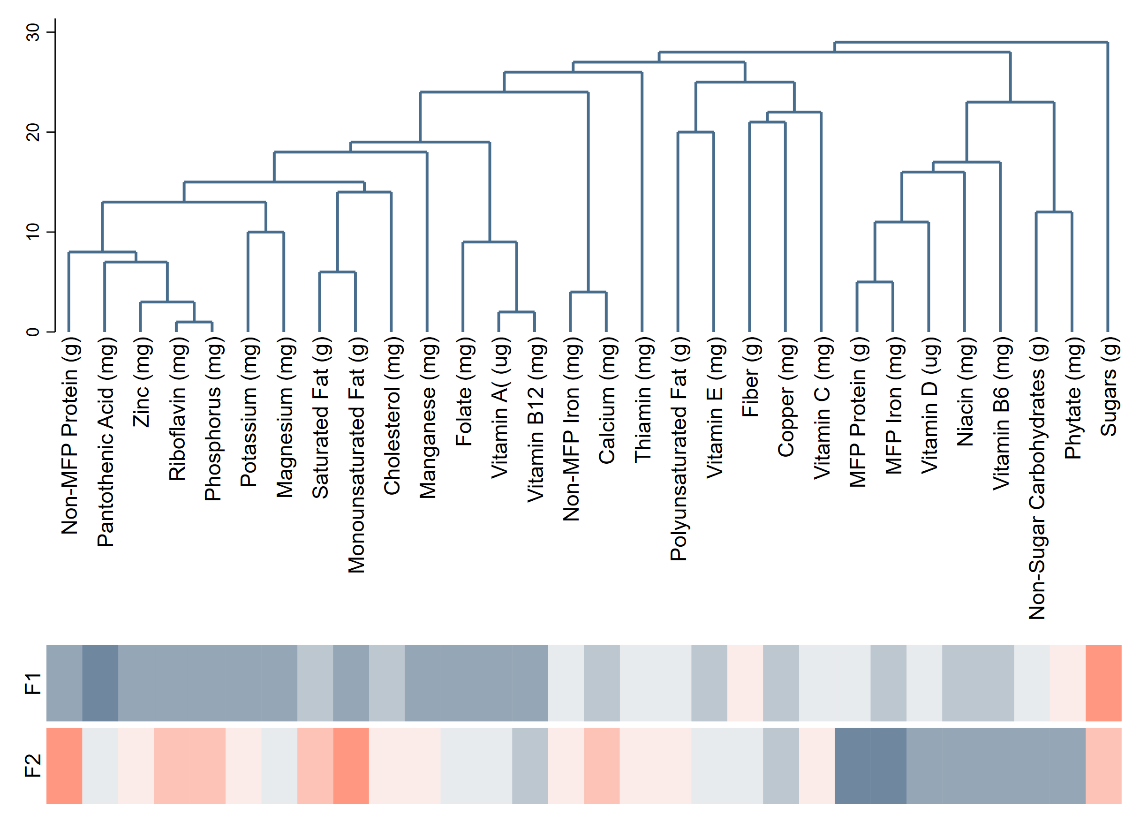 |
| --- |
| 1. **Dendrogram of PCA-Derived Dietary Factors. (Child Data)**   The treelet dendrogram is provided to aid in interpretation  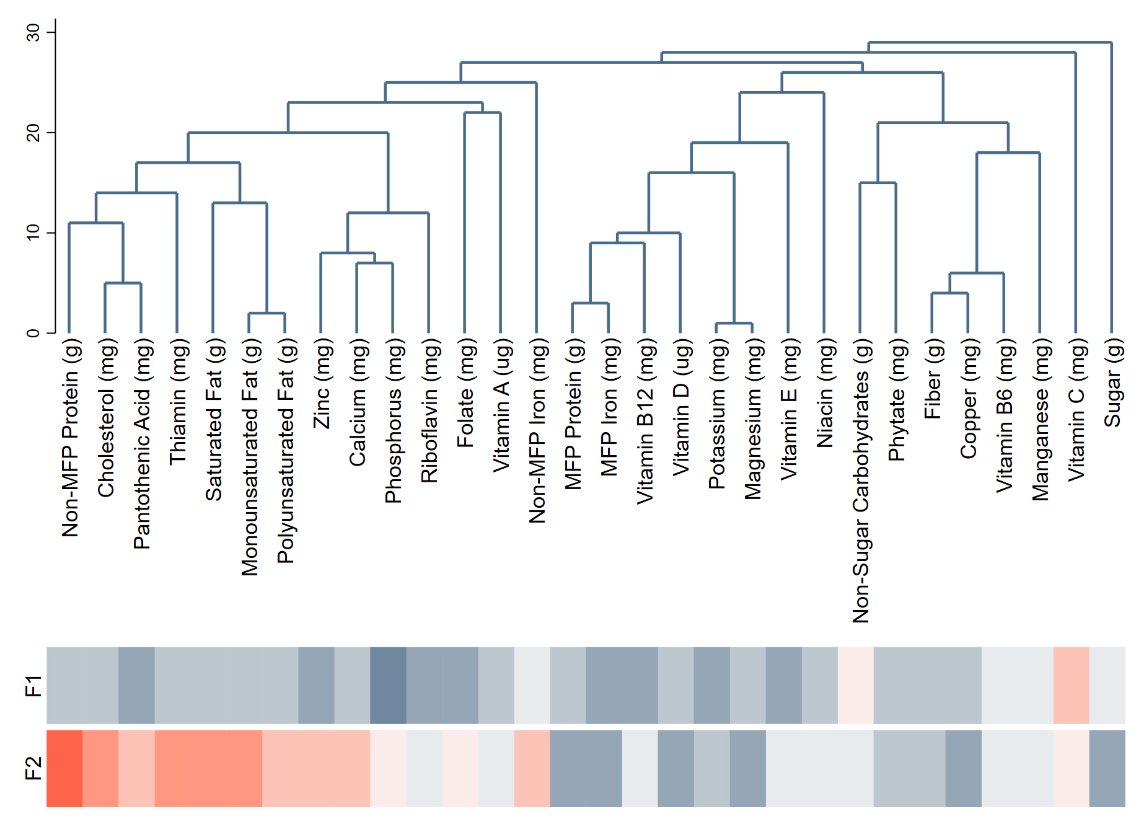 |

**Supplemental Figure 3.** **PCA-Derived Child Dietary Factors**

| **a. Forest Plot of Associations between PCA-Derived Dietary Factors and Cardiometabolic biomarkers**  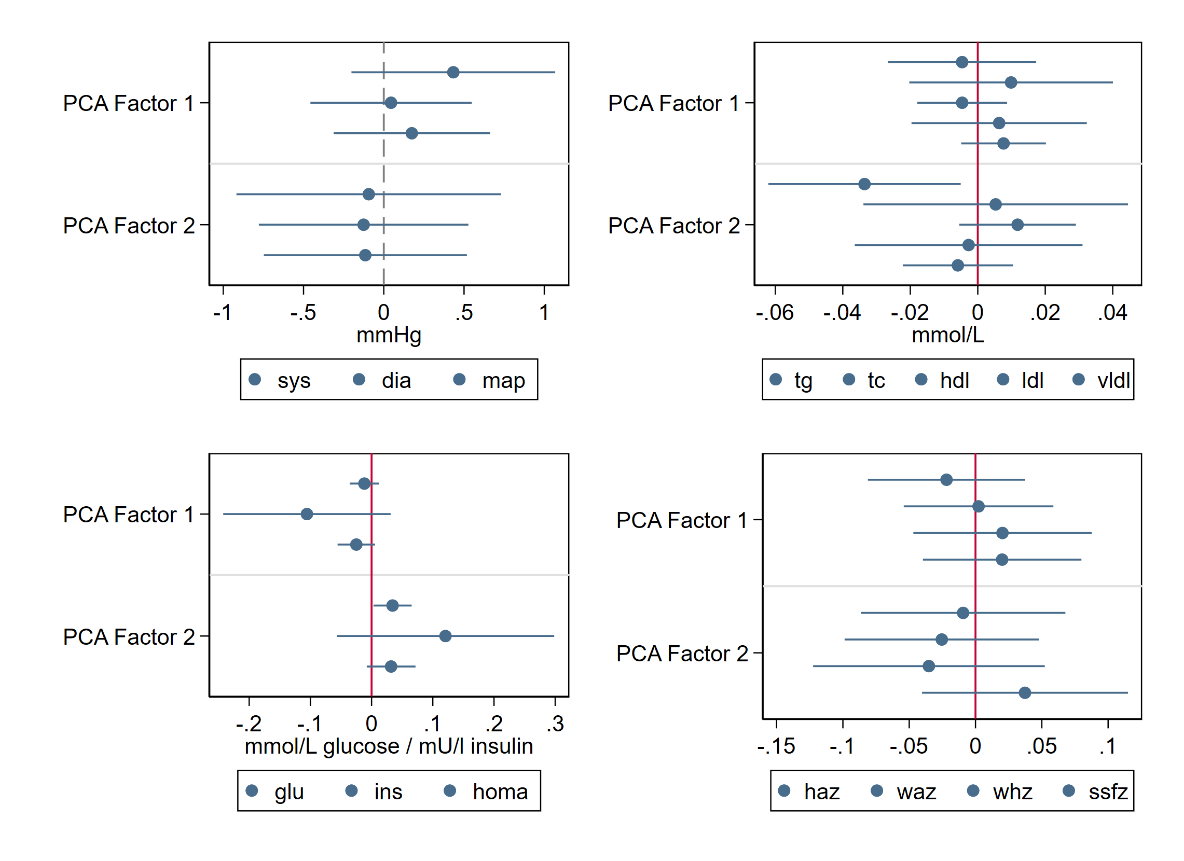 |
| --- |
| **b. Forests plots of associations between PCA-derived factors and cardiometabolic outcomes.**  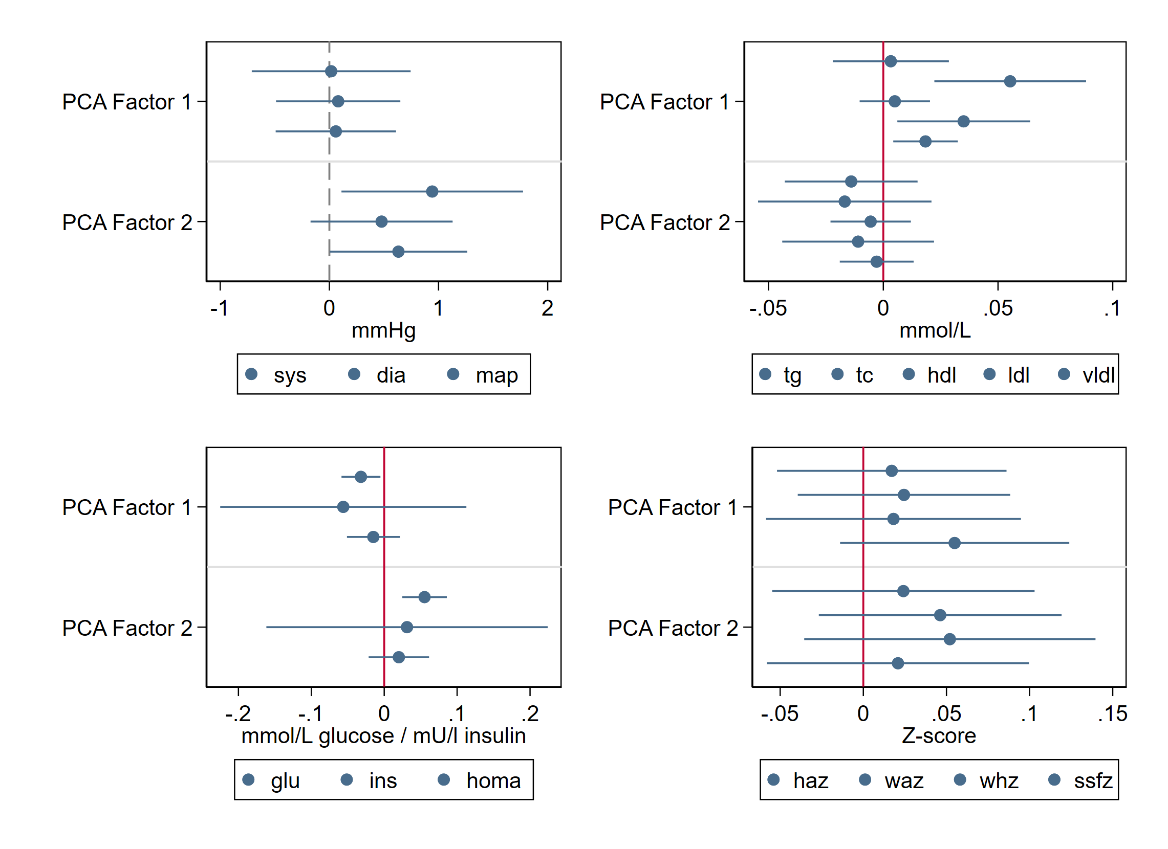 |
